# Cellular dynamics of EMT: lessons from live in vivo imaging of embryonic development

**DOI:** 10.1186/s12964-021-00761-8

**Published:** 2021-07-22

**Authors:** Jeffrey D. Amack

**Affiliations:** 1 Department of Cell and Developmental Biology, State University of New York Upstate Medical University, Syracuse, NY USA; 2 BioInspired Syracuse: Institute for Material and Living Systems, Syracuse, NY USA

**Keywords:** Epithelial-mesenchymal transition (EMT), Mesenchymal-epithelial transition (MET), Cell migration, Cancer metastasis, Actin cytoskeleton, In vivo live imaging, Embryonic development, Gastrulation, Neural crest cell development, Kupffer’s vesicle

## Abstract

Epithelial-mesenchymal transition (EMT) refers to a process in which epithelial cells lose apical-basal polarity and loosen cell–cell junctions to take on mesenchymal cell morphologies and invasive properties that facilitate migration through extracellular matrix. EMT—and the reverse mesenchymal-epithelial transition (MET)—are evolutionarily conserved processes that are used throughout embryonic development to drive tissue morphogenesis. During adult life, EMT is activated to close wounds after injury, but also can be used by cancers to promote metastasis. EMT is controlled by several mechanisms that depend on context. In response to cell–cell signaling and/or interactions with the local environment, cells undergoing EMT make rapid changes in kinase and adaptor proteins, adhesion and extracellular matrix molecules, and gene expression. Many of these changes modulate localization, activity, or expression of cytoskeletal proteins that mediate cell shape changes and cell motility. Since cellular changes during EMT are highly dynamic and context-dependent, it is ideal to analyze this process in situ in living organisms. Embryonic development of model organisms is amenable to live time-lapse microscopy, which provides an opportunity to watch EMT as it happens. Here, with a focus on functions of the actin cytoskeleton, I review recent examples of how live in vivo imaging of embryonic development has led to new insights into mechanisms of EMT. At the same time, I highlight specific developmental processes in model embryos—gastrulation in fly and mouse embryos, and neural crest cell development in zebrafish and frog embryos—that provide in vivo platforms for visualizing cellular dynamics during EMT. In addition, I introduce Kupffer’s vesicle in the zebrafish embryo as a new model system to investigate EMT and MET. I discuss how these systems have provided insights into the dynamics of adherens junction remodeling, planar cell polarity signaling, cadherin functions, and cytoskeletal organization during EMT, which are not only important for understanding development, but also cancer progression. These findings shed light on mechanisms of actin cytoskeletal dynamics during EMT, and feature live in vivo imaging strategies that can be exploited in future work to identify new mechanisms of EMT and MET.

**Video Abstract**

**Supplementary Information:**

The online version contains supplementary material available at 10.1186/s12964-021-00761-8.

## Background

Embryonic development depends on epithelial cells changing into migratory mesenchymal cells, and then changing back into epithelial cells when they reach their destination. These interlinked cellular dynamics, termed epithelial-mesenchymal transition (EMT) and mesenchymal-epithelial transition (MET), have long been recognized as fundamental processes that drive development [1]. In adult tissues, EMT is involved in wound healing in response to injury [2]. However, prolonged EMT activation caused by chronic inflammation can lead to fibrosis and scar formation. In addition, during the process of metastasis, cancer cells can active EMT to break away from an epithelial tumor, migrate and invade a new tissue, and then undergo MET to seed a new tumor [3]. Thus, understanding EMT and MET may provide insight into underlying causes of developmental malformations that can lead to birth defects, mechanisms of fibrosis, and how cancer spreads throughout the body.

**Fig. 1:**
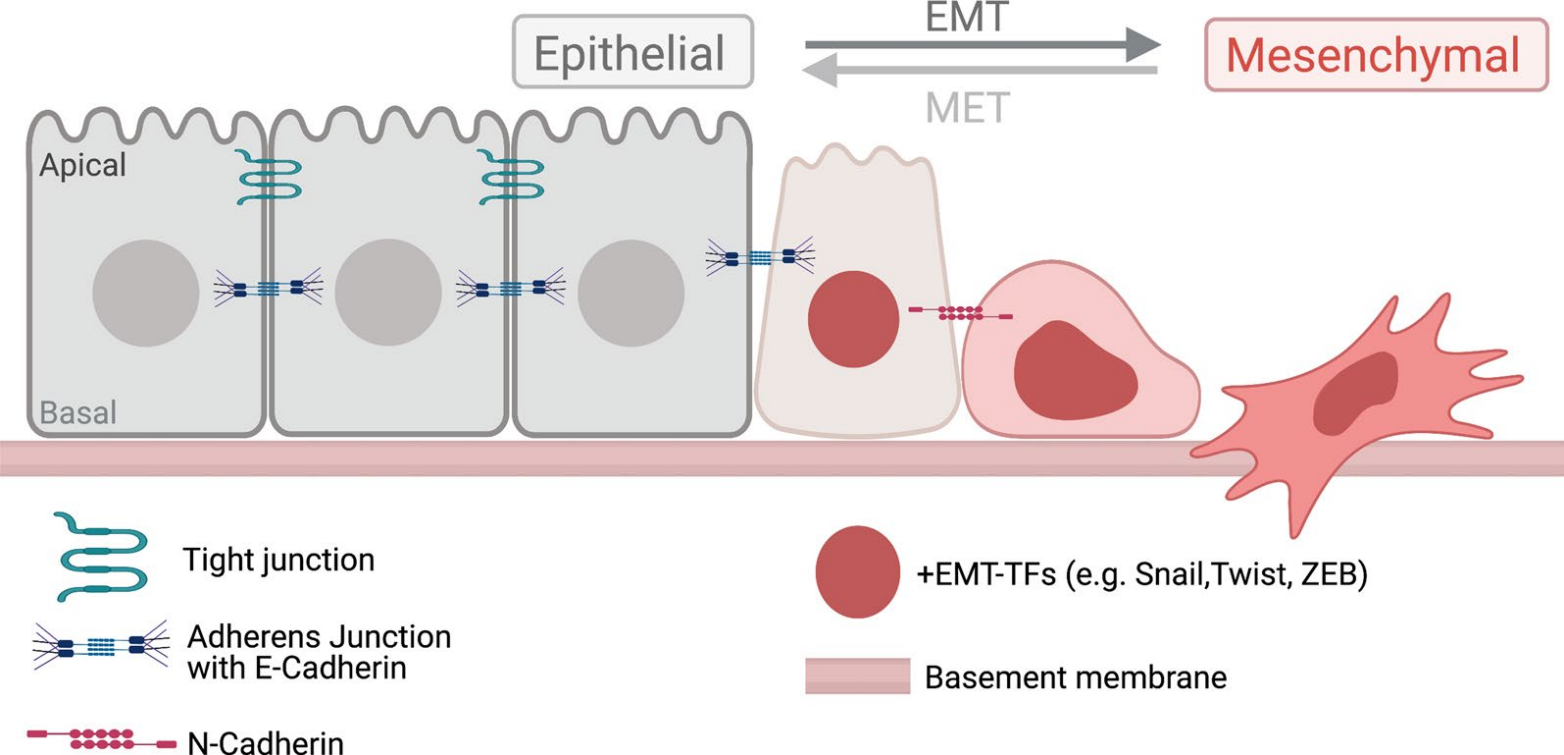
Overview of EMT and MET transitions. Epithelial-mesenchymal transition (EMT) is a dynamic process in which cells turn on EMT-promoting transcription factors (EMT-TFs), disassemble cell–cell junctions, lose apical-basal polarity, and upregulate new cadherins. Cells also undergo extensive rearrangements of actin cytoskeleton that mediate shape changes, front-rear polarity, invasive behavior, and migration. In many cases in vivo, EMT is partial and cells have both epithelial and mesenchymal properties. A mesenchymal-epithelial transition (MET) is the reverse process

The process of EMT is characterized by several key events that change cell adhesion, cell shape and cell motility (Fig. 1). Specific details and molecular mechanisms of these steps have been reviewed previously (examples include: [4–7]), so I provide here only a brief overview. It is clear that multiple cell–cell signaling pathways—including TGFβ, Wnt, Notch, and FGF—work in context-dependent ways to induce changes in epithelial cells by, in part, activating EMT-promoting transcription factors (EMT-TFs). EMT-TFs in the Snail, Twist, and ZEB families repress epithelial genes and activate mesenchymal genes. As one example, Snail directly represses expression of epithelial cadherin (E-cadherin), a transmembrane component of adherens junctions (AJs) that form between epithelial cells. AJs are protein complexes that mediate cell–cell adhesion, and interact with the actin cytoskeleton to provide mechanical links between cells in a tissue. Disassembly of AJs is a critical step that allows cells undergoing EMT to delaminate, or detach, from an epithelium and become migratory. EMT-TFs also downregulate expression of epithelial polarity proteins, which leads to loss of apical-basal polarity and disassembly of tight junctions. Non-canonical Wnt/planar cell polarity signaling plays a critical role in regulating mesenchymal cell migration during development and cancer progression [8, 9]. These cells develop front-rear polarity driven by rearrangements of the actin cytoskeleton, which results in the formation of actin-based protrusions—lamellipodia and filopodia—that mediate cell migration [6]. Actin-rich structures called invadopodia are involved in proteolytic degradation of extracellular matrix that facilitates invasion through epithelial basal lamina or basement membrane [10]. Actin dynamics during EMT are regulated by several signaling molecules, including Rho family GTPases [11]. In general, Rac1 and Cdc42 control actin polymerization and protrusion formation at the leading edge, whereas Rho regulates cell retraction at the trailing edge. In addition, Rho can activate Rho-associated kinase to phosphorylate Myosin II regulatory light chain, which enhances actomyosin contractility that controls tension and cell shape. Mechanisms and regulators of the dynamic reorganization of the actin cytoskeleton during EMT and subsequent mesenchymal cell migration are discussed in previous review articles [11–14].

Recent work in both development and cancer indicates that EMT is typically not an all-or-nothing switch between epithelial and mesenchymal cell types. Instead, EMT is a dynamic process with intermediate states in which cells have both epithelial and mesenchymal properties [15–17]. This ‘partial’ EMT maintains cell–cell contacts that allow cells to move collectively. Evidence from developing embryos and cancer metastases indicates both single cell migration and collective cell migration can occur downstream of EMT in vivo [18, 19]. Since the progression of EMT encompasses so many cellular dynamics, which highly depend on local environmental cues, it is ideal to study this process as it occurs in vivo. Microscopy of living embryos has been used for decades to provide insights into highly dynamic developmental processes. Live imaging is an important approach in biology because, in some cases, live imaging of cellular dynamics has uncovered new and surprising results that were not anticipated from analyzing snapshots of fixed samples [20]. Over the years, live imaging approaches in embryos have included using differential interference contrast microscopy to follow cells and tissues, using fluorescent dyes to label individual cells, and more recently, expressing genetically encoded fluorescent tagged proteins in specific cell types. Time-lapse imaging of living embryos allows quantitative analysis of cell behaviors and sub-cellular events, in many cases, at very high spatial and temporal resolution [21]. In the case of EMT, live imaging can capture the dynamic behaviors of cells as they change shape, delaminate, and begin to migrate. Indeed, mesenchymal cell migration is a direct output—and useful readout—of EMT that can be tracked and measured over time. It is important to note that live imaging of isolated cells, tissue explants, tumors, and organoids have made important contributions to our understanding of EMT, but I focus here on insights from time-lapse imaging of developmental processes in living embryos.

The overarching goals of this review are to (1) discuss recent lessons learned from live imaging of embryonic development that contribute to our understanding of actin cytoskeletal dynamics during EMT in vivo, and (2) describe how live imaging approaches in model embryos are used to visualize cellular dynamics (see Table 1). This is not an exhaustive review, but rather highlights selected recent works that provide interesting new insights into regulation and/or functions for the actin cytoskeleton during specific EMT cellular processes—AJ remodeling, planar cell polarity signaling, cadherin functions, and cytoskeletal organization—that are not only important for understanding development, but each of these is also implicated in cancer progression. The selected live imaging approaches focus primarily on two developmental programs—gastrulation and neural crest cell development—that are established paradigms for investigating EMT during embryo development. First, gastrulation is a crucial stage of early animal development in which embryonic epithelial cells undergo EMT and migrate to form the three germ layers of cells, endoderm, mesoderm, and ectoderm, that give rise to all cell types in the body [22, 23]. Second, neural crest cells undergo EMT to delaminate from the epithelium of the neural tube and migrate long distances in characteristic streams of cells that can include single cells and collective cell migration [24]. In addition to these established platforms, I propose Kupffer’s vesicle in the zebrafish embryo as a model system to study MET-EMT. Mesenchymal dorsal forerunner cells and epithelial Kupffer’s vesicle cells undergo a tightly controlled series of EMT-MET-EMT transitions that are essential for left–right patterning of the embryo [25, 26]. In future work, new innovations in live imaging using these systems are expected to continue to provide new surprises and new mechanistic insights into the cellular dynamics of EMT and MET in vivo.

**Table 1:** Examples of insights into EMT gained from in vivo live imaging of embryonic development

Major findings	Developmental process	Model Embryo	Live imaging strategy	Microscopy	References
Actomyosin contractility promotes AJ remodeling and protects AJs from Snail-dependent disassembly during EMT	Gastrulation	*Drosophila*	Label AJs with GFP-tagged E-Cad Detect Myosin II activity using mCherry-tagged Myosin II RLC	Laser scanning confocal	[32]
FGF signaling regulates AJ dynamics and cell division during EMT, and apical-basal polarization during MET	Gastrulation	*Drosophila*	Label nuclei with GFP-tagged Histone 2A	Multi-photon	[42]
Pk1 deficient NCCs fail to transition to mesenchymal morphology, and have altered Cadherin expression	Neural crest cell development	Zebrafish	Express GFP using NCC-specific promoter Label F-actin in NCCs with Lifeact:GFP	Laser scanning confocal	[55]
Pk1-mediated PCP signaling regulates adhesion forces between mesenchymal cells during MET	Kupffer’s vesicle development	Zebrafish	Express GFP using DFC/KV promoter	Multi-photon	[67]
PCP-mediated migration regulates mechanical stiffness of mesoderm that induces NCC EMT	Neural crest cell development	*Xenopus*	Transplant fluorescent NCCs Measure mesoderm elastic modulus	Compound fluorescence Atomic force microscopy	[73]
Cdh6 regulates the spatial distribution of Rho GTPase activity that localizes F-actin and apical detachment during EMT	Neural crest cell development	Zebrafish	Express membrane localized GFP using NCC-specific promoter Label F-actin in NCCs with mCherry-UtrCH Use Rho biosensor to measure Rho activity	Laser scanning confocal	[85]
Crb2 regulates AJ disassembly and actomyosin-driven EMT	Gastrulation	Mouse	Mosaic label cells with Cre-mediated activation of membrane-GFP	Laser scanning confocal	[91]
After EMT, mesodermal subpopulations develop different cell morphologies, migration dynamics, and cytoskeletal compositions	Gastrulation	Mouse	Mosaic label cells with Cre-mediated activation of membrane-GFP Label F-actin in with Lifeact:GFP	Multi-photon	[98]

### Insights into the regulation of adherens junctions during EMT

Disassembly of adherens junctions (AJs) between cells is a critical step of EMT. Live imaging of fluorescent-tagged AJ-associated proteins in developing *Drosophila* (fruit fly) embryos provides a powerful approach to investigate AJ dynamics and AJ-mediated cell biology. The *Drosophila* embryo begins as a large syncytium, and then undergoes cellularization to give rise to epithelial cells. During gastrulation, presumptive mesoderm cells undergo cell shape changes, internalization, EMT, and migration [22]. In this case, EMT is a long process, during which AJs remodel and gradually disassemble. When mesoderm cells are finished migrating, they form a monolayer and undergo MET. Due to some interesting developmental differences between Drosophila and vertebrates, the *Drosophila* embryo provides an opportunity to investigate mechanistic drivers of EMT-MET and associated cellular behaviors that might be missed in vertebrate embryos. Here, I highlight two examples of how live imaging of mesoderm during *Drosophila* gastrulation have shed light on AJ dynamics and the mechanisms that regulate them. The first study reveals specific functions for Snail, Myosin II, and actomyosin contractility in AJ remodeling and disassembly, and the second study uncovers roles for FGF signaling that regulates AJs, cell division, and apical-basal polarity during EMT and MET. Importantly, these two studies capture cell behaviors during intermediate stages of EMT, which are difficult to visualize and interpret without live imaging.

Snail family transcription factors are known to promote AJ disassembly by mediating transcriptional repression of the AJ component E-cadherin during both embryo development and cancer progression [27, 28]. During EMT in *Drosophila* gastrulation, AJ disassembly in mesoderm cells is a Snail-dependent process. Interestingly, although Snail represses E-cadherin mRNA expression, there is plenty of maternally supplied E-cadherin protein when AJs disassemble. This indicates Snail also mediates posttranscriptional mechanisms that promote AJ disassembly [29]. Curiously, AJ dynamics do not directly correlate with Snail expression in mesodermal epithelial cells in the pre-gastrula *Drosophila* embryo. Although Snail protein is expressed in early ventral mesoderm, AJs do not immediately disassemble. Instead, AJs reorganize from subapical sites to form tight apical puncta [30, 31], and then disassemble only after the cells internalize during gastrulation. Changes in the actin cytoskeleton are implicated in AJ remodeling, and AJ disassembly, but the precise mechanisms that control AJ dynamics during gastrulation EMT are not completely understood.

**Fig. 2:**
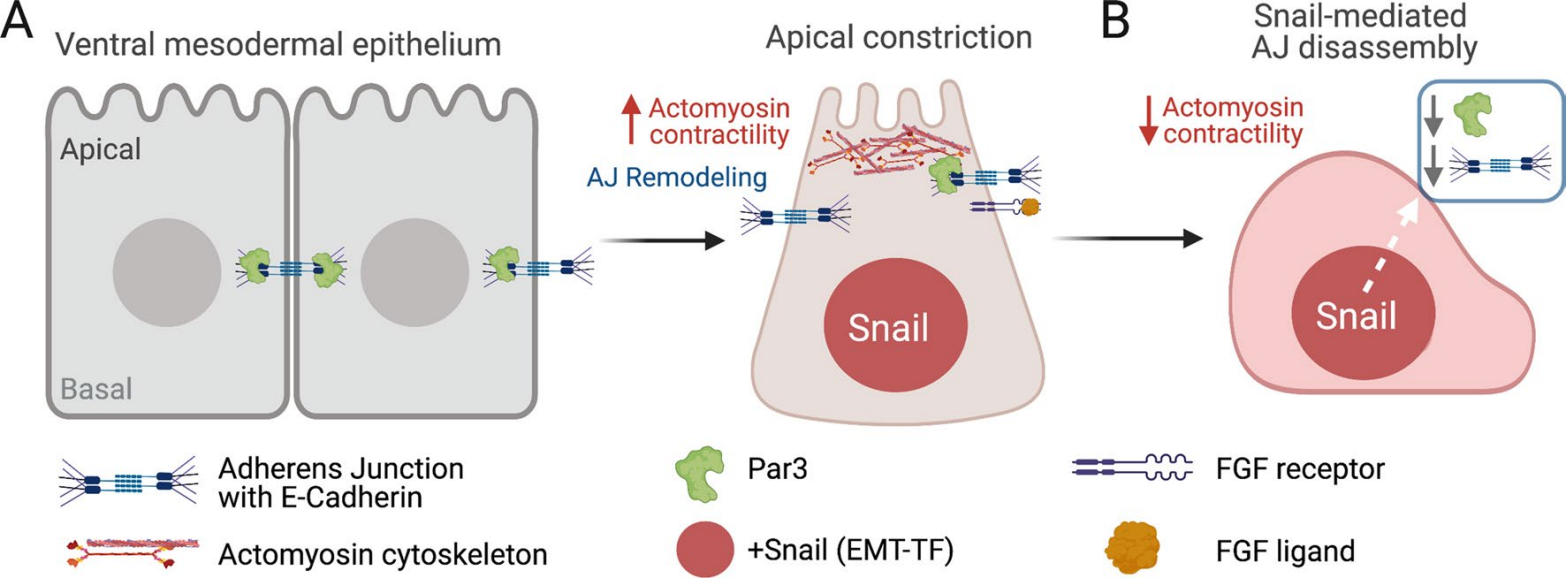
Actomyosin contractility and FGF signaling regulate adherens junction dynamics during *Drosophila* gastrulation. **A** During apical constriction of ventral mesoderm cells, increased contraction of the actin-myosin cytoskeleton mediates remodeling of adherens junction (AJ) complexes from sub-apical to apical positions. The polarity protein Par3 localizes to remodeled AJs, and Myosin II-generated tension protects AJs from Snail-mediated disassembly [32]. FGF receptor (Heartless) localizes with apical AJs, and FGF signaling modulates AJ number and cell division (not shown) during gastrulation and EMT [42]. **B** As Myosin II levels decrease, Snail activity leads to Par3 downregulation and AJ disassembly to promote EMT [32, 37]

Using live confocal microscopy to visualize AJs during gastrulation, Weng and Weischaus [32] found that actomyosin activity mediates AJ reorganization in mesodermal epithelial cells during gastrulation, and delays Snail-mediated AJ disassembly. Using GFP-tagged E-cadherin [33] to visualize AJs in live embryos, in combination with genetic perturbations, the authors found that existing subapical AJs move apically to form apical junctions, and that this process depends on actomyosin contractility (Fig. 2A). AJ remodeling may involve lateral clustering and/or vesicle trafficking of E-cadherin, which are known to be regulated by the actomyosin cytoskeleton [34, 35]. Visualization of mCherry-tagged Myosin II regulatory light chain [36] revealed that Myosin II accumulates at AJs during *Drosophila* gastrulation. Reduction of Myosin II levels delayed AJ remodeling in ventral mesoderm cells, and activation of Myosin II in dorsal cells induced AJ remodeling. These results are consistent with AJs remodeling in response to increased cortical tension that occurs during cell shape changes. In addition, actomyosin contractility was found to antagonize Snail-mediated AJ disassembly. These findings indicate that increased contractility promotes AJ remodeling and protects AJ integrity from post-transcriptional Snail-dependent disassembly. Interestingly, Snail expression leads to downregulation of apical-basal polarity protein Par3 (called Bazooka in fly) that localizes to remodeled apical AJs strengthened by Myosin II (Fig. 2B). In follow-up work, the authors suggest removal of Par3 from AJs—mediated by Snail upregulation and/or reduced actomyosin contractility—could contribute to junction disassembly [37].

In addition to understanding the cytoskeletal controls of EMT, there is great interest in identifying signaling pathways that regulate specific steps of EMT. The fibroblast growth factor (FGF) signaling pathway has been extensively studied in development, and has been found to regulate multiple cellular behaviors [38]. In the mouse embryo, FGF is an upstream regulator of Snail expression that mediates E-cadherin transcriptional repression to drive EMT during gastrulation [39]. In contrast, EMT during gastrulation in *Drosophila* can occur in the absence of FGF signaling [40]. However, live imaging of mesoderm in *Drosophila* FGF mutants identified defects during migration and a failure to organize into a monolayer after migrating [40, 41]. Thus, the roles for FGF signaling in this process are unclear.

To better understand functions for FGF signaling during *Drosophila* gastrulation, Sun and Stathopoulus [42] genetically altered FGF signaling and then followed mesoderm cell migration by imaging fluorescent nuclei labeled with GFP-tagged Histone 2A [43]. Tracking cell movements and quantifying angular cell positions revealed that cells over-expressing FGF ligand failed to undergo dorsal movements, which are observed in wild-type cells. Additional analyses suggested ectopic FGF increases the number of AJs. Immunostaining experiments indicate the FGF receptor Heartless (Htl) localizes apically with AJs during mesoderm EMT (Fig. 2A). Live imaging of nuclei also revealed that FGF signaling regulates the rate of cell division, which is proposed to contribute to EMT during gastrulation by decreasing cell–cell adhesion. Finally, FGF was found to regulate apical accumulation of cell polarity proteins, including Par3/Bazooka, in mesoderm cells during monolayer formation and MET. Importantly, Snail protein levels were not affected by FGF loss-of-function or gain-of-function, indicating these phenotypes are independent of Snail transcription. This work reveals new roles for FGF signaling in AJ dynamics and cell division during EMT, and apical-basal polarization during MET. It would be interesting to test in future work whether FGF signaling mediates changes in actomyosin activity to control AJ dynamics and cell polarization.

### Insights into how PCP signaling generates biophysical cues to impact EMT and MET

Planar cell polarity (PCP) refers to a polarized orientation of cells across a plane of a tissue. Genetic screens in *Drosophila* first identified molecules that are required for polarization of actin-rich hair-like structures on epithelial cells in the wings (reviewed in [44]). Core PCP components localize to AJs at specific membrane domains to establish proximal–distal asymmetry in epithelial cells: Frizzled (Fz), Disheveled (Dsh) and Diego (Dgo) proteins localize to distal cell–cell junctions, whereas Van Gogh (Vang) and Prickle (Pk) localize proximally [45]. Intercellular interactions between these PCP components organize polarization of the cells in the epithelium. In vertebrate embryos, conserved core PCP signaling components set up planar polarity in several epithelia [46, 47]. In addition, PCP signaling regulates several cell movements in the embryo, including convergence and extension movements of the mesoderm during gastrulation [8, 48]. Loss of PCP signaling disrupts mediolateral polarity of mesodermal cells and how they intercalate. Vertebrate PCP signaling is a non-canonical (β-catenin-independent) Wnt pathway mediated by specific Wnt ligands that include Wnt5 and Wnt11 [49]. PCP signaling can activate downstream effectors—including Rho GTPases—that modulate actin cytoskeletal dynamics to control cell behaviors [50]. Dysregulation of non-canonical Wnt/PCP signaling has been reported in several tumor types and is associated with promoting cancer metastasis [9, 51]. In this section, I provide an example for how live imaging of neural crest cells in zebrafish have identified functions for the PCP component Pk1 in regulating actin dynamics and cell shape changes during EMT. Next, I introduce the zebrafish Kupffer’s vesicle as a model system to study EMT and MET, and discuss evidence for Pk1 and PCP mediating cell–cell adhesion forces during MET. Finally, I review work in *Xenopus* that indicates PCP regulates cell movements that create mechanical stiffness of mesoderm to induce neural crest cell migration. These studies present new mechanistic links between PCP signaling and cell shape changes, cell–cell-adhesion, and tissue stiffness that only live imaging could provide.

Detachment of neural crest cells (NCCs) from the neuroepithelium in vertebrate embryos is an extensively studied process to understand mechanisms of EMT and subsequent mesenchymal cell migration [18, 24]. NCCs migrate throughout the embryo to take up residence and contribute to several different tissues and organs. This normal developmental process has been compared to the process of cancer metastasis [52]. In zebrafish, the frog *Xenopus laevis*, and chicken embryos, PCP signaling is required for NCC migration [53]. During NCC migration, the core PCP proteins Dsh and Fz mediate contact inhibition of locomotion, which occurs when NCCs come in physical contact and then migrate apart [54]. However, functions for PCP components during NCC EMT are not fully understood.

**Fig. 3:**
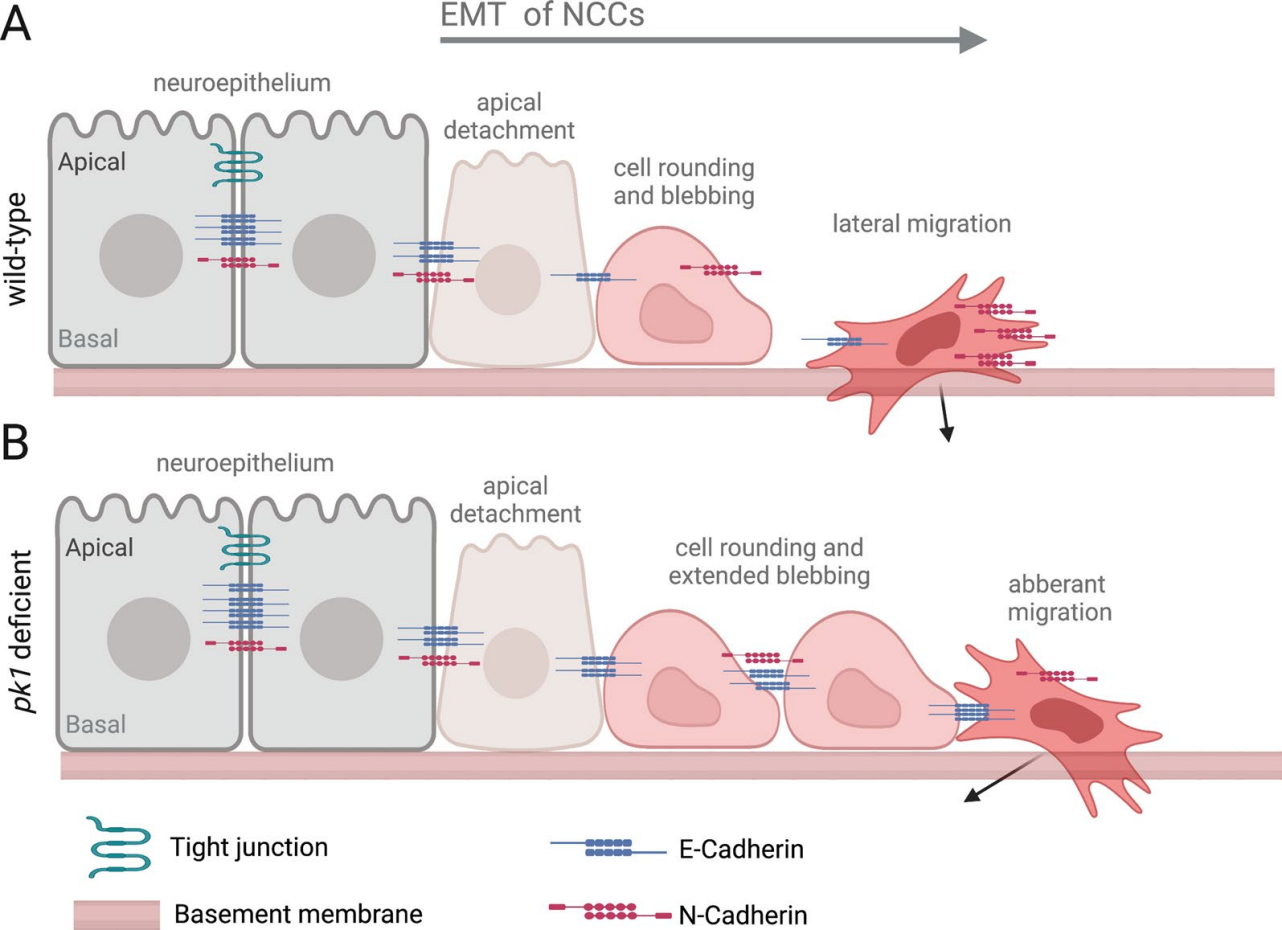
Planar cell polarity protein Prickle 1 mediates the transition of neural crest cells to a mesenchymal morphology. **A** Wild-type pre-migratory neural crest cells (NCCs) in the zebrafish neuroepithelium undergo EMT behaviors that include detachment from the apical surface, cell rounding and membrane blebbing at the basal surface, and a transition to protrusive mesenchymal morphology for lateral migration. **B** In embryos with a mutation in the core PCP Prickle 1 genes *pk1a* or *pk1b*, NCCs cluster and undergo abnormal anterior migration along the neural tube [55]. These mutant NCCs bleb for an extended period and largely fail to transition to mesenchymal morphology. Some NCCs that do become mesenchymal and migratory fail to separate from adjacent NCCs. Knockdown of Pk1b results in an increase of E-cadherin and a decrease of N-cadherin in migratory NCCs as compared to wild-type

Working with zebrafish embryos, Ahsan, et al. [55] used genetics and live imaging to test the function of the core PCP *prickle 1* (*pk1*) genes in NCC development. In *Drosophila*, Pk is a cytoplasmic protein that is recruited to proximal membrane of polarized cells by interacting with Vang. The zebrafish genome contains two *pk1* genes, *pk1a* and *pk1b*, and their functions are not well understood. During EMT, zebrafish NCCs detach from the apical midline of the neuroepithelium, round up at the basal neuroepithelium, initiate membrane blebbing, and then delaminate and become protrusive and migratory [56] (Fig. 3A). In contrast to wild-type cranial NCCs that successfully complete EMT and transition to an elongated morphology and migrate in well-characterized streams, NCCs in *pk1a* or *pk1b* mutants were largely found to remain rounded and clustered at the neural tube. To visualize NCCs in live *pk1* mutant embryos, expression of the *Tg(sox10:EGFP)* transgene [57], which labels all NCCs with EGFP, was used to track cranial NCCs for 2 h in embryos at 16 h post-fertilization (hpf) when NCCs are finishing EMT and beginning to migrate. Calculating displacement trajectories of individual cells revealed that instead of normal lateral movement of NCCs out of the neuroepithelium, *pk1a* or *pk1b* mutant NCCs retained cell–cell contacts and moved in an anterior direction (Fig. 3B). Next, expression of a *Tg(sox10:Lifeact-GFP)* transgene [58] was used to label actin-rich filopodia and lamellipodia in NCCs. Following detachment from the neuroepithelium, wild-type NCCs quickly transition from a rounded morphology that forms blebs to a mesenchymal state that is highly protrusive and migratory (Fig. 3A). Pk1 deficient NCCs detached normally, but then maintained an extended blebbing behavior and many cells failed to transition to a mesenchymal morphology (Fig. 3B). Mutant NCCs that did become mesenchymal failed to detach from neighboring cells. These aberrant NCC behaviors correlate with immunostaining results that show high levels of E-cadherin and low levels of neural cadherin (N-cadherin) in *pk1b* deficient NCCs relative to wild-type siblings. This suggests Pk1b mediates NCC EMT by regulating the levels of cell adhesion molecules, potentially via a PCP-mediated feedback loop. Since PCP signaling can activate Rho GTPase-mediated changes in the actin cytoskeleton, it would be interesting to determine whether Pk1 regulates cytoskeletal dynamics that alter AJs and/or actin organization in NCCs to promote the transition from blebbing round cells to protrusive mesenchymal cells.

**Fig. 4:**
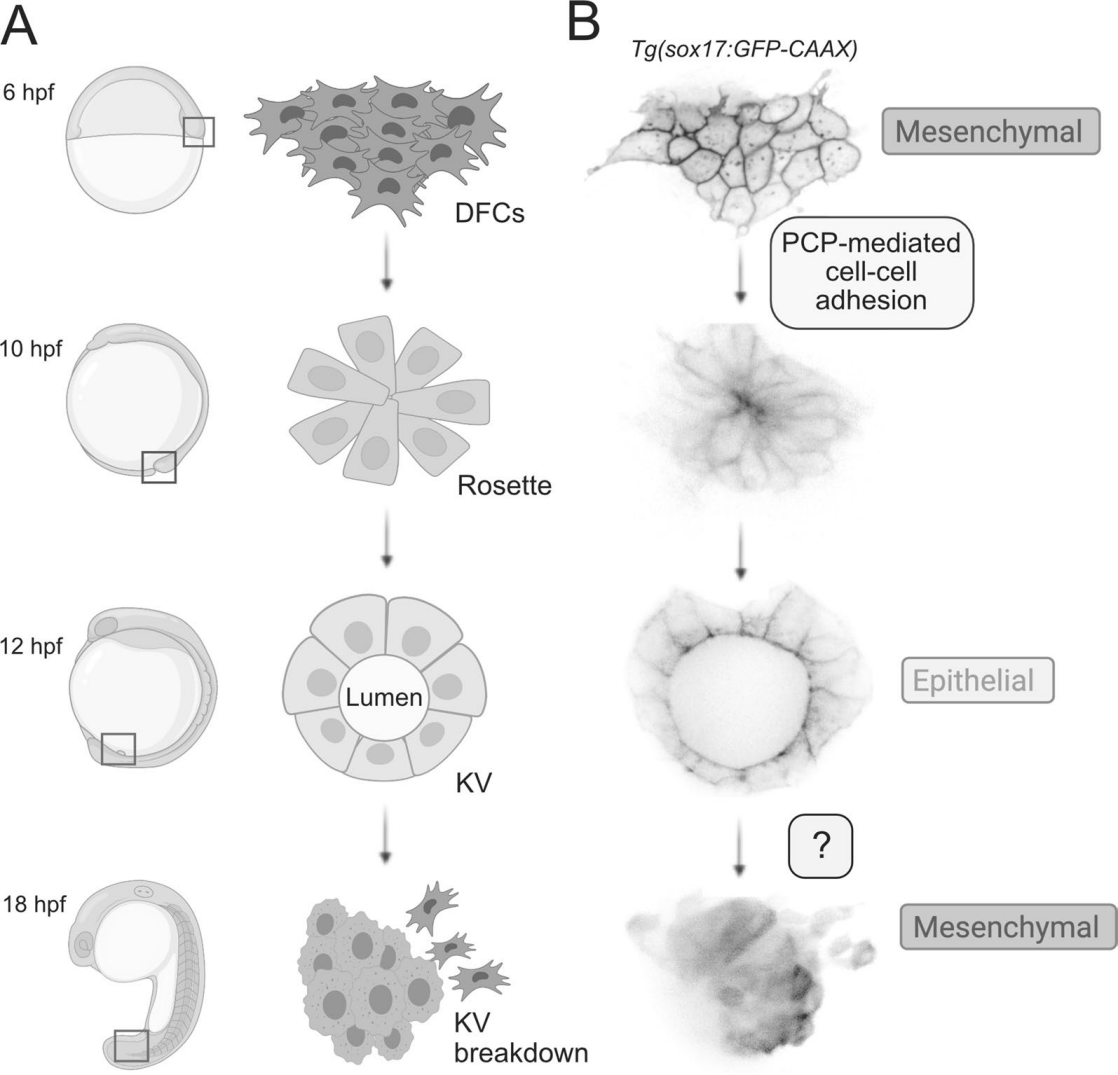
Development of the zebrafish Kupffer’s vesicle as a model to investigate mechanisms that control EMT and MET. **A** Schematic of Kupffer’s vesicle development in zebrafish. Boxes on embryo diagrams on the left indicate location of cells depicted on the right. At 6 h post-fertilization (hpf), precursor cells called dorsal forerunner cells (DFCs) are specified at the dorsal margin. Mesenchymal DFCs migrate and then form a rosette structure at 10 hpf. DFCs undergo MET to form the epithelial Kupffer’s vesicle (KV). A cross section through the KV depicts epithelial cells lining a fluid-filled lumen at 12 hpf. After KV functions to establish the left–right body axis, KV collapses at 18 hpf and the epithelial cells undergo EMT and migrate away. **B** Confocal microscopy images of live transgenic *Tg(sox17:GFP-CAAX)* embryos that express membrane-targeted GFP in DFC and KV cells at developmental stages corresponding to diagrams in A. The planar cell polarity (PCP) proteins Prickle 1a (Pk1a) and Wnt11 regulate cell–cell adhesion between DFCs during MET and rosette formation [67]. Mechanisms that control KV breakdown and EMT of KV cells remain unknown

In addition to regulating morphology and adhesion of NCCs during EMT, Pk1 has been implicated in regulating cell–cell adhesion forces between mesenchymal cells called dorsal forerunner cells (DFCs) in the zebrafish embryo. DFCs give rise to the transient epithelial organ Kupffer’s vesicle (KV) that functions to orient the left–right body axis of the embryo [59, 60]. Development of the DFC/KV cell lineage presents an attractive and largely untapped model system to uncover mechanisms that control EMT and MET. Several transgenic zebrafish strains that express green or red fluorescent proteins in DFC/KV cells have been generated to facilitate live imaging experiments [61–64], and a Cre-based mosaic labeling approach has been developed to quantify 3-dimensional behaviors and morphometrics of individual DFC/KV cells during development [64]. Additional advantages of this experimental system include easy access to externally fertilized embryos, optical transparency of the embryos, and rapid development of KV. Live imaging experiments using two-photon or spinning disc confocal microscopy of DFC/KV cells labeled in transgenic strains, including *Tg(sox17:GFP-CAAX)* strain that expresses plasma membrane targeted GFP in DFC/KV cells [64], has defined multiple steps of DFC/KV development (Fig. 4). First, the specification of DFCs is controlled by TGFβ/Nodal signaling that instructs epithelial enveloping layer cells to transition into mesenchymal DFCs [26]. DFCs then collectively migrate along of the dorsal edge of the embryo, and ultimately coalesce to form a rosette structure and undergo MET to form epithelial KV cells [25, 26]. The ball of KV cells, which surround a fluid-filled lumen that expands over time, project motile cilia into the lumen that beat to create an asymmetric fluid flow to establish left–right asymmetric gene expression. Once left–right patterning of the embryo is established, live imaging shows that the KV organ breaks down and KV cells undergo EMT and migrate away to incorporate into notochord, somites, and tail tissues [65, 66]. Live imaging by our group has captured the process of KV breakdown (Additional file 1: Movie 1), which has not been previously studied and provides a new opportunity to probe in vivo mechanisms of EMT. Based on these strengths in live imaging methods, in combination with the ability to engineer gene mutations and conduct large-scale genetic and drug screens in zebrafish, I propose KV as a useful model system to investigate mechanisms of MET and EMT.

To investigate the function of PCP signaling in DFC/KV development, Oteiza, et al. [67] used mutants, gene knockdowns, and live imaging of *Tg(sox17:GFP)* transgenic embryos [61] that express GFP in the DFC/KV lineage. In contrast to control embryos, in which DFCs migrate and cluster to form a tight rosette structure during MET, live imaging showed that DFCs in embryos deficient for Pk1a and Wnt11 failed to coalesce into a single rosette. Further analyses indicated that a normal number of mesenchymal DFCs was specified and migrated normally in PCP deficient embryos, but the number of epithelial cells incorporated into the KV was reduced, which resulted in the formation of a smaller KV lumen. Using a DFC-specific knockdown approach [68], the authors found that Pk1a functions cell-autonomously in DFCs to control KV development. Innovative in vitro studies using single cell force spectroscopy to quantify cell–cell adhesion properties of isolated DFCs indicated the adhesion force between DFCs was reduced by Pk1a knockdown. Single cell force spectroscopy results also measured a reduced tether force in Pk1a deficient DFCs, which suggests a defect in membrane tension. Disrupted membrane tension may alter the clustering of adhesion proteins at the cell surface, which could explain DFC adhesion defects in Pk1a knockdown cells. These results suggest PCP-mediated adhesion forces are necessary for mesenchymal DFCs to successfully transition into epithelial KV cells (Fig. 4B). Interestingly, in addition to cell–cell adhesion forces between DFCs, the authors speculate that mechanical interactions between DFCs and the overlying enveloping layer may contribute to DFC rosette formation and epithelial KV development.

In addition to zebrafish, work in several other models have contributed to our understanding of how biophysical forces and mechanical properties of cells and tissues are integrated with biochemical signaling to drive morphogenesis during embryo development [69, 70]. Cell behaviors are influenced by biophysical geometry and material properties of the local environment. During migration, cells can sense their environment to respond to a gradient of substrate-bound cues in the extracellular matrix, which is called haptotaxis, and/or a gradient of tissue stiffness (durotaxis) [71]. Previous work has identified PCP signaling as a good candidate for modulating the mechanical properties of tissues via regulation of cell adhesion, extracellular matrix and/or cell movements [72].

Intriguing work in *Xenopus* suggests stiffness of head mesoderm provides biophysical cues that induce migration of NCCs possibly by triggering EMT. Mechanistically, PCP-mediated convergence and extension movements increase mechanical stiffness of mesoderm that lies underneath the neural crest. In this work, Barriga, et al. [73] used tissue grafts to transplant a fluorescent labeled neural crest to an unlabeled donor, and then used time-lapse imaging to watch NCCs migrating in the frog embryo. In addition, the authors applied a different type of ‘live imaging’ by using atomic force microscopy to measure stiffness of mesoderm tissue in living *Xenopus* embryos. Atomic force microscopy was used to measure apparent elastic modulus of head mesoderm that serve as the substrate for NCCs migration. Mesoderm stiffness increased between developmental stages when NCCs are non-migratory (stage 13) and pre-migratory (stage 20). In vivo laser ablations and gene knockdowns revealed that reducing mesoderm stiffness stopped NCC migration, suggesting changes in tissue mechanical properties function as a trigger for migration. Moreover, using atomic force microscopy to increase stiffness induced premature NCC migration. In vivo manipulations indicated that extracellular matrix and actomyosin contractility are not essential for mesoderm stiffness. Instead, stiffness increases as convergence and extension movements of migrating mesoderm increase cell density of the head mesoderm. Inhibiting PCP signaling with the Disheveled mutant Dsh-DEP [74] disrupts convergence and extension and thereby reduces cell density. This decreased mesoderm stiffness and blocked initiation of NCC migration. Adding extrinsic compression using atomic force microscopy rescued NCC migration in PCP signaling deficient embryos. These results provide a new mechanical link between PCP-mediated migration of mesoderm during gastrulation and initiation of migration of overlying NCCs. It is proposed that changes in mesoderm stiffness trigger NCC migration by promoting EMT. A mechanical cue for EMT is an exciting concept, but it should be noted that the experiments here focused on post-EMT NCC migration and did not directly determine whether stiffness impacts the process of EMT. It remains possible that mechanisms that regulate NCC EMT are separable from mechanisms that regulate NCC migration away from the neural tube.

### Insights into Cadherin 6 functions during EMT

During EMT, cadherins undergo dynamic changes in expression (referred to as ‘cadherin switching’) that are mediated by both transcriptional and post-transcriptional mechanisms. For example, NCCs express E-cadherin, N-cadherin, and Cadherin 6 (also known as Cad6B in chick) prior to EMT, and then N-cadherin and Cadherin 6 are downregulated while Cadherin 7 and Cadherin 11 are upregulated in migrating NCCs [24]. Downregulation of chick Cadherin 6 via Snail-mediated repression and post-transcriptional controls is necessary for NCCs to complete EMT, but the function(s) for Cadherin 6 during EMT are not fully understood. In the chick embryo, Cadherin 6 knockdown reduces the number of migrating trunk NCCs due to failed EMT [75], suggesting Cadherin 6 functions to promote EMT. In cranial NCCs, Cadherin 6 depletion just before EMT promotes premature migration [76], suggesting Cadherin 6-mediated adhesion inhibits NCC EMT. These findings suggest Cadherin 6 molecules have context-specific roles during EMT. In addition to regulating development, Cadherin 6 is implicated in cancer metastasis [77, 78]. Here, I review insights into Cadherin 6 functions during NCC EMT in chick embryos, and then discuss results from live imaging of NCCs in zebrafish that suggest Cadherin 6 regulates Rho GTPase activity and F-actin during NCC detachment. Prior to live imaging experiments, the dynamics of Rho GTPases and the actin cytoskeleton during NCC EMT were unknown.

Valuable insights into Cadherin 6 regulation and function during EMT have come from analyzing NCCs in the chick embryo. Recently, new high-resolution live imaging strategies have been developed to track and quantify NCC behaviors in the living chick embryo, but studies thus far have focused on NCC migration dynamics rather than EMT mechanisms [79–81]. However, experiments using in vivo manipulations of Cadherin 6, followed by immunostaining of NCC markers, provide evidence that protein fragments generated from the proteolytic cleavage of Cadherin 6 function as regulators of EMT. During Cadherin 6 downregulation, the Cadherin 6 protein is cleaved by ADAM10, ADAM19 and γ-secretase [82]. Intracellular cleavage by γ-secretase creates C-terminal fragments that associate with transcriptional regulator β-catenin in the cytoplasm of NCCs and can enter the nucleus with β-catenin to upregulate expression of pro-EMT genes, including the EMT-TF *Snail2* [83]. N-terminal Cadherin 6 fragments generated by ADAMs-mediated cleavage in the extracellular domain promote EMT by increasing the degradation of fibronectin and laminin extracellular matrix in the neural tube basement membrane [84]. These studies indicate Cadherin 6 has multiple roles during EMT.

**Fig. 5:**
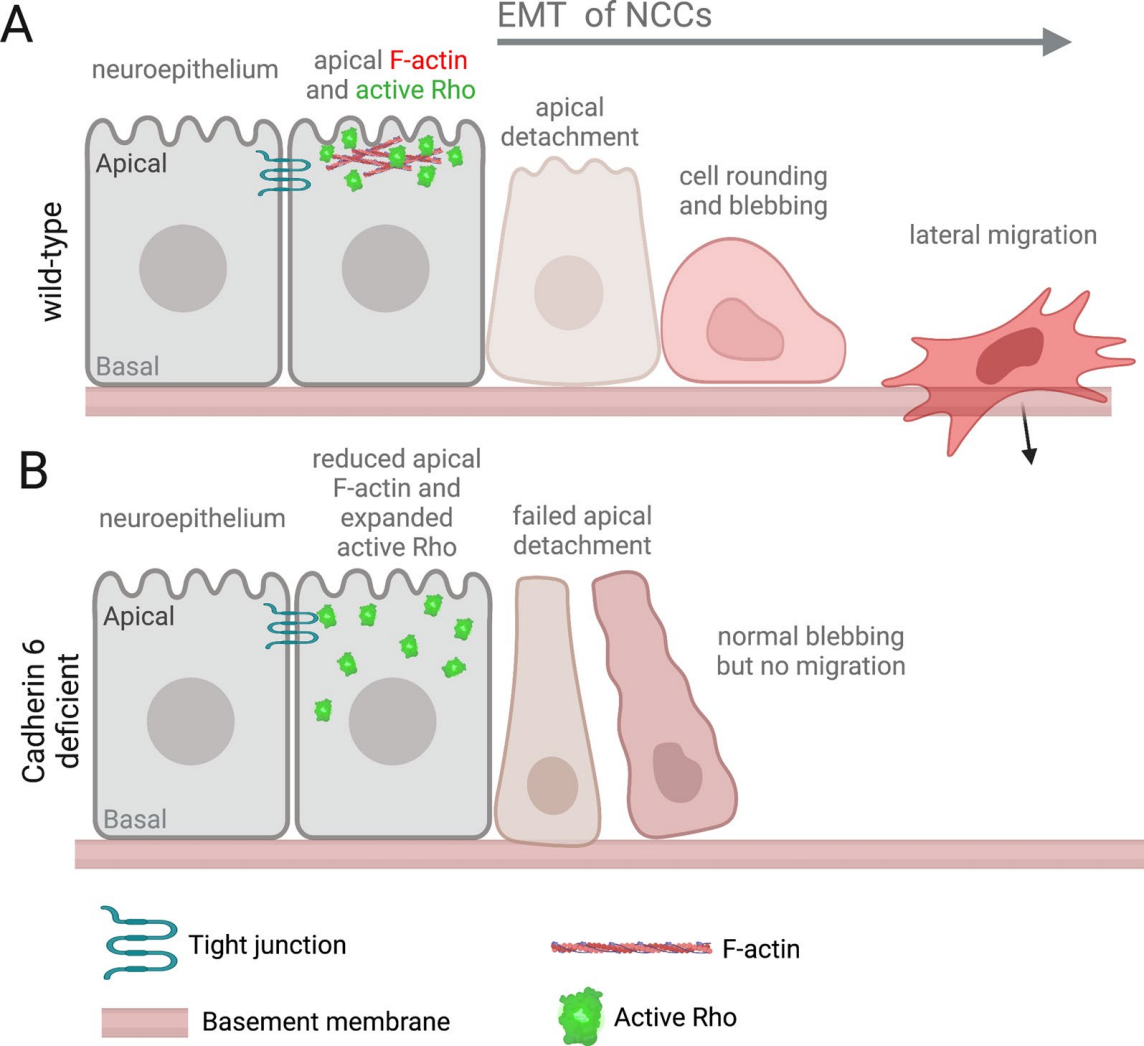
Cadherin 6 regulates active Rho GTPase distribution, F-actin accumulation, and apical detachment in zebrafish neural crest cells during EMT. **A** Wild-type zebrafish pre-migratory neural crest cells (NCCs) show an apical accumulation of active Rho GTPases and filamentous actin (F-actin), which are necessary for actomyosin-mediated apical detachment and subsequent lateral migration during EMT. **B** In most Cadherin 6 knockdown NCCs, F-actin fails to accumulate in the apical tail, active Rho is more widely distributed, the apical tail does not detach, and the cells do not undergo EMT or initiate migration [85]

To test the functions of Cadherin 6 during NCC EMT in living embryos, Clay and Halloran [85] created mosaically labeled NCCs in zebrafish embryos by microinjecting plasmid DNA encoding a *Tg(sox10:GFP-CAAX)* transgene [86] that expresses membrane localized GFP specifically in NCCs. Live imaging of individual NCCs starting at 14 hpf revealed that Cadherin 6 knockdown blocked NCCs from detaching from the neuroepithelium at the apical midline, one of the earliest steps of EMT. In a previous live imaging study, these authors found that F-actin accumulation and Rho GTPase activity are critical for actomyosin-mediated NCC apical detachment [86]. To test whether Cadherin 6 regulates F-actin accumulation in NCCs, the fluorescent F-actin binding protein mCherry-UtrCH [87] was used to assess F-actin dynamics. In wild-type NCCs, F-actin accumulated in the apical trailing/tail region of NCCs just prior to detachment and migration (Fig. 5A). In Cadherin 6 knockdown embryos, F-actin failed to accumulate in the tail region in most NCCs, and these cells did not undergo EMT (Fig. 5B). Interestingly, a subset of Cadherin 6 knockdown NCCs had normal apical accumulation of F-actin, and these cells successfully completed EMT. Together, these results indicate Cadherin 6 function mediates apical F-actin accumulation in NCCs during EMT. Next, to determine whether Cadherin 6 impacts Rho activity in NCCs, the Rho biosensor GFP-rGBD (GFP fused to the RhoA binding domain of Rhotekin) [88] was used to visualize Rho GTPase activity in live imaging experiments. Cadherin 6 knockdown did not alter the level or timing of Rho activation in NCCs, but did result in an expansion of Rho activity within a cell (Fig. 5B). Together, these results suggest Cadherin 6 regulates the spatial distribution of Rho GTPase activity and F-actin accumulation in NCCs and subsequent apical detachment and EMT. It remains to be seen whether these functions are mediated by proteolytically cleaved fragments of Cadherin 6.

### Insights into cytoskeletal organization before and after EMT

As discussed above, EMT that occurs during gastrulation has been studied via live imaging of the fly embryo, which has provided insights into mechanisms of EMT and cell migration. More recently, advances in microscopy platforms and embryo culture protocols have made it feasible to image gastrulation in living mouse embryos. During mouse gastrulation, epithelial epiblast cells undergo EMT to migrate through the primitive streak and form mesoderm and endoderm [23]. EMT, which initiates at the primitive streak, depends on basement membrane breakdown, apical constriction and basal positioning of the nucleus in the epiblast cell, and loss of AJs. Expression of the transcription factor Sox2 is downregulated and Snail1 expression is upregulated. Cells that successfully undergo EMT delaminate from the epithelium and begin to migrate in a process called ingression. Recent live imaging studies have begun to uncover cellular behaviors and cytoskeletal dynamics during mouse gastrulation, which cannot be studied using fixed specimens. I discuss two examples of how live imaging in mouse has revealed unexpected insights into regulation of cytoskeletal organization associated with EMT. First, work on the membrane protein Crumbs2 indicates Myosin II localization promotes cell ingression, and second, differential cell morphology and migration dynamics in mesoderm subpopulations reflect different cytoskeleton compositions that emerge as outcomes of EMT.

Several signaling molecules have been identified as regulators of gastrulation EMT in mouse, but how these signals control distinct cell behaviors is not completely understood. For example, genetic analysis revealed that knockout of *Crumbs2* (*Crb2*) causes mesoderm defects and embryonic lethality mid-gestation due to defects in gastrulation [89]. Crumbs proteins are transmembrane molecules that have a large extracellular domain and a smaller intracellular domain that contains conserved protein–protein interaction domains that can associate with components of signaling pathways [90]. Mice have 3 *Crumbs* genes, but only *Crb2* is essential for embryogenesis [91]. In *Drosophila*, the single Crumbs protein is involved in organizing apical-basal polarity in epithelial cells [92]. In contrast, mouse embryos with mutations in *Crumbs* genes establish normal apical-basal polarity during gastrulation [91]. This suggests distinct and unknown functions for Crumbs proteins during mouse development.

**Fig. 6:**
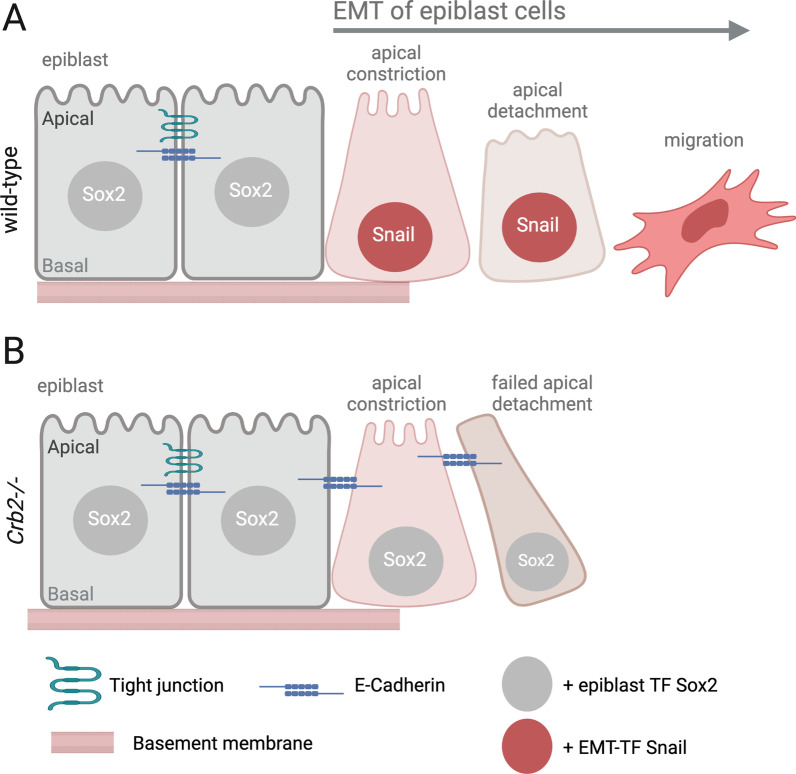
Crumbs2 mediates adherens junction disassembly and apical detachment of epiblast cells as they undergo EMT during gastrulation in the mouse embryo. **A** During gastrulation, wild-type epiblast cells near the primitive streak undergo EMT that involves basement membrane breakdown, apical constriction and basal positioning of the nucleus, and loss of E-cadherin containing adherens junctions (AJs). EMT correlates with downregulation of the transcription factor (TF) Sox2 and upregulation of the EMT-TF Snail1. Cells that successfully undergo EMT detach from the epithelium and migrate into the primitive streak in a process called ingression. **B** In *Crumbs2* mutant (*Crb2-/-*) embryos, mosiacally labeled epiblast cells undergo apical constriction and basal nuclear displacement, but fail to detach from the epithelium [91]. *Crb2-/-* cells develop an elongated morphology and remain attached to the epithelium via E-cadherin. Apical Myosin II accumulation is reduced in *Crb2-/-* epiblast relative to wild-type (not shown), suggesting a link between Crb2 and actomyosin activity during EMT [91]

To gain insight into how loss of Crb2 impacts mouse gastrulation, Ramkumar, et al. [91] used live microscopy to follow individual labeled cells in developing mouse embryos. Cells were mosaically labeled using Cre-mediated activation of membrane-localized GFP in the mT/mG transgenic reporter line [93]. mT/mG embryos ubiquitously express the membrane-targeted red fluorescent protein Tomato (mT), which can be switched to membrane-targeted green fluorescent protein (mG) by Cre-mediated excision. Mosaic fluorescent labeling of the cell membrane can provide an outline of single cells and facilitate quantitative morphological analyses. The *EIIA-Cre* strain [94] was used to mosaically label individual cells during gastrulation, which were imaged in cultured embryos on embryonic day 7.5 (E7.5) for 4–6 h. Live imaging revealed that during cell ingression, wild-type epiblast cells undergo apical constriction, basal positioning of the cell body, and then detach from the epithelium (Fig. 6A). *Crb2-/-* mutant cells also show apical constriction and basal cell body displacement—indicators that EMT is initiated—but the cells don’t exit the epithelium (Fig. 6B). Analyses of cell morphology and immunostaining indicate that.

*Crb2-/-* cells have abnormal elongated cell shapes, are Sox2 positive, and remain attached to the epithelium via thin protrusions that contain E-cadherin protein. Together these results indicate *Crb2-/-* epiblast cells fail to disassemble AJs and fail to complete EMT. Additional work indicates Crb2 is involved in apical accumulation of Myosin II in mouse epiblast poised to undergo ingression, and inversely correlates with Myosin II localization. These results support a model in which Crb2 regulates actomyosin-driven cell ingression during EMT, and may provide insight into how polarity complexes promote cancer metastasis [95]. Future work is needed to determine mechanistically how Crb2 integrates with the actomyosin cytoskeleton to control EMT.

In addition to identifying regulatory molecules during EMT, work has focused on understanding outcomes of gastrulation in the mouse embryo. During gastrulation EMT and epiblast cell ingression, subpopulations of mesoderm emerge: the embryonic mesoderm gives rise to tissues in the embryo, and the extra-embryonic mesoderm contributes to chorion, amnion and yolk sac [96]. Little is known about mechanisms that regulate behaviors of these subpopulations. However, genetic analysis indicates the Rho family GTPase Rac1 is required for embryonic mesoderm migration, but not extra-embryonic mesoderm [97]. Since Rac1 is a known regulator of the actomyosin cytoskeleton, this implicates cytoskeletal dynamics in regulating differential behaviors of mesoderm subtypes. Live imaging provides a powerful approach to investigate the dynamics of distinct cell populations as they emerge following EMT.

Similar to the approach taken by Ramkumar, et al. [91] described above, Saykali, et al., [98] used Cre-mediated labeling of mesoderm cells in mT/mG reporter mice to track and quantify cell behaviors over developmental time. Embryos were isolated on E6.75 or E7.25 for live imaging for up to 12 h. Cells destined to form embryonic mesoderm had a different morphology than presumptive extra-embryonic mesoderm cells. Embryonic mesoderm cells were smaller and had more cell protrusions/filopodia per cell than extra-embryonic mesoderm cells. In addition, cells that form embryonic mesoderm migrated from anterior to posterior following a zigzag path, whereas extra-embryonic mesoderm lacked overall directionality and moved slower. Live imaging of mesoderm-specific knockout of the Rho GTPases Rac1 or RhoA revealed reduced migration speed of embryonic mesoderm, but not extra-embryonic mesoderm. This indicates embryonic mesoderm depends on Rac1 and RhoA for migration, whereas extra-embryonic mesoderm does not. Consistent with these results, phalloidin staining and live imaging of the fluorescent F-actin binding protein LifeAct-GFP indicates embryonic mesoderm cells have dense F-actin networks, whereas extra-embryonic cells do not. In contrast, the intermediate filament Keratin 8 is enriched in extra-embryonic mesoderm. Together, these results show that mesodermal subpopulations have different cell morphologies, migration dynamics, and cytoskeletal compositions. During cancer progression, EMT can result in a range of context-dependent mesenchymal phenotypes [7]. This work provides an in vivo example of cells that take on different cytoskeletal and behavioral phenotypes that can be differentially regulated downstream of EMT.

## Conclusions

EMT and MET are fundamental biological processes that are essential for normal embryonic development. Not surprisingly, dysregulation of EMT can result in malformations of tissues and organs that are underlying causes of disease [1, 99]. Cancer progression can also involve EMT and MET. There are certainly differences between cancer EMT and developmental EMT, but there are also similarities. In many cases, lessons learned about EMT from embryos have been applicable to understanding cancer progression [52]. This review presents examples of insights into EMT gained from live imaging of embryonic development. Focusing largely on mechanisms mediated by the actin cytoskeleton, these insights include functions for Crb2 and actomyosin activity in regulating AJ dynamics, involvement of PCP signaling in regulating cell shape, cell adhesion and tissue stiffness, and a role for Cadherin 6 regulating local Rho activity and actin dynamics. This review also discusses the imaging strategies that were used to generate these findings. The fly, zebrafish, frog, and chicken embryos discussed here offer well-established systems to visualize and quantify dynamic cellular processes such as EMT. In addition, recent advances provide opportunities to image developing mouse embryos in vivo. It is also important to note that high resolution live imaging is feasible in other model systems not described here, including but not limited to *Caenorhabditis elegans* and *Dictyostelium discoideum*, that provide alternative systems to investigate cell behaviors and mechanisms that may be relevant to cancer progression [100].

Advances in light microscopy are expected to continue to push the limits of temporal and spatial resolution of cellular and subcellular processes in vivo. In future studies, live imaging of in vivo EMT has potential to shed light on a number of outstanding questions. For example, several recent studies have focused on the question of whether metastasis, in specific contexts, is mediated by single circulating tumor cells or clusters of cells [101]. In contrast to single cell dynamics, circulating tumor cell clusters are predicted to exhibit collective migration behaviors [102]. Live imaging of developmental processes offers opportunities to investigate context-dependent mechanisms that regulate both single cell and collective cell migration. In addition, fluorescent fusion proteins and/or activity sensors that monitor cellular dynamics (e.g. AJs, EMT-TFs, actin dynamics) could be used combination to further understand ‘partial EMT’ phenotypes as they develop in real time and in different microenvironments. Also, while mechanisms that induce EMT are well studied, the regulators of MET are not well defined. Live imaging of developmental processes, including the zebrafish KV discussed here, may provide new insight on the biochemical and/or mechanical signals that trigger migrating mesenchymal cells to transition into an epithelium. Taken together, the continued development of innovative live in vivo imaging strategies in embryonic systems will provide new opportunities to test specific hypotheses and identify new regulators of cellular dynamics that drive EMT and MET.

## Supplementary Information

**Additional file 1: Movie 1**. Live imaging of transgenic Tg(sox17:GFP-CAAX) embryos with fluorescently labeled Kupffer’s vesicle (KV) cells that have been pseudo-colored green. As the KV organ breaks down, epithelial undergo EMT and migrate away. Time stamp: hrs:min.

## Data Availability

Not applicable.

## Abbreviations

EMT: Epithelial-mesenchymal transition
MET: Mesenchymal-epithelial transition
AJ: Adherens junctions
PCP: Planar cell polarity
TGFβ: Transforming growth factor beta
FGF: Fibroblast growth factor
EMT-TF: EMT-promoting transcription factor
GFP: Green fluorescent protein
NCC: Neural crest cell
EGFP: Enhanced green fluorescent protein
DFC: Dorsal forerunner cell
KV: Kupffer’s vesicle
ADAM: A disintegrin and metalloprotease domain
mT/mG: Membrane-targeted tandem dimer Tomato (mT)/membrane-targeted green fluorescent protein (mG)
F-actin: Filamentous actin
Hpf: Hours post-fertilization
